# Establishment of Human PD-1/PD-L1 Blockade Assay Based on Surface Plasmon Resonance (SPR) Biosensor

**DOI:** 10.21769/BioProtoc.4765

**Published:** 2023-08-05

**Authors:** Tess Puopolo, Huifang Li, Justin Gutkowski, Ang Cai, Navindra P. Seeram, Hang Ma, Chang Liu

**Affiliations:** Department of Biomedical and Pharmaceutical Sciences, College of Pharmacy, University of Rhode Island, Kingston, RI 02881, USA;

**Keywords:** PD-1, PD-L1, Blockade, Surface plasmon resonance, Cancer, Immunotherapy

## Abstract

Blockade of the programmed cell death protein 1 (PD-1)/PD-ligand 1 (PD-L1) axis is a promising strategy for cancer immunotherapy. Although antibody-based PD-1/PD-L1 inhibitors have shown remarkable results in clinical cancer studies, their inherent limitations underscore the significance of developing novel PD-1/PD-L1 inhibitors. Small molecule inhibitors have several advantages over antibody-based inhibitors, including favorable tumor penetration and oral bioavailability, fewer side effects, easier administration, preferred biological half-life, and lower cost. However, small molecule inhibitors that directly target the PD-1/PD-L1 interaction are still in the early development stage, partially due to the lack of reliable biophysical assays. Herein, we present a novel PD-1/PD-L1 blockade assay using a surface plasmon resonance (SPR)-based technique. This blockade assay immobilizes human PD-1 on a sensor chip, which interacts with PD-L1 inhibitors or negative PD-L1 binders with human PD-L1 protein at a range of molecular ratios. The binding kinetics of PD-L1 to PD-1 and the blockade rates of small molecules were determined. Compared to other techniques such as PD-1/PD-L1 pair enzyme-linked immunosorbent assay (ELISA) and AlphaLISA immunoassays, our SPR-based method offers real-time and label-free detection with advantages including shorter experimental runs and smaller sample quantity requirements.

Key features

A SPR protocol screens compounds for their capacity to block the PD-1/PD-L1 interaction.

Validation of PD-1/PD-L1 interaction, followed by assessing blockade effects with known inhibitors BMS-1166 and BMS-202, and a negative control NO-Losartan A.

Analysis of percentage blockade of PD-1/PD-L1 of the samples to obtain the IC_50_.

Broad applications in the discovery of small molecule–based PD-1/PD-L1 inhibitors for cancer immunotherapy.

Graphical overview

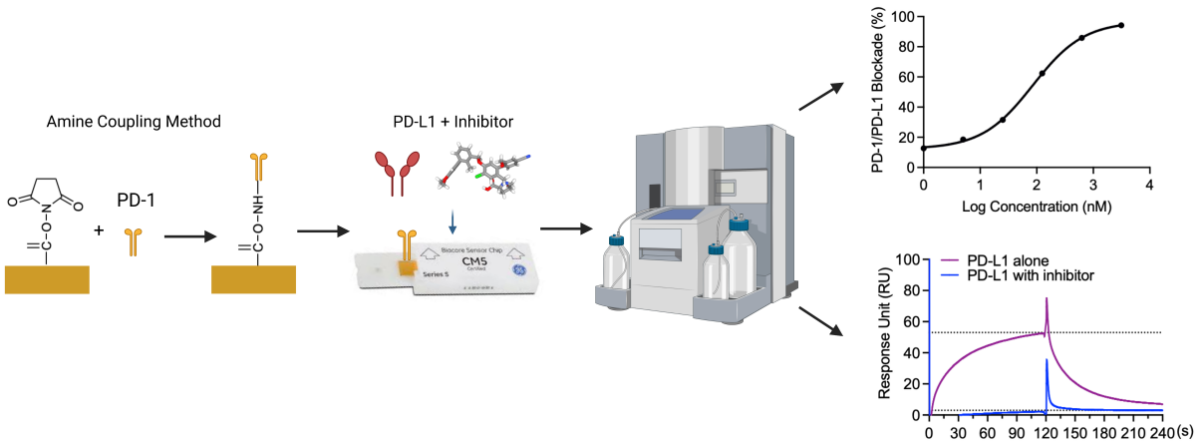

## Background

Cancer is a global health burden resulting in high healthcare costs. Therefore, the search for effective therapeutics is of continued scientific interest. Recently, cancer immunotherapy, a strategy that utilizes the host’s own immune system to fight tumors, has become an effective treatment of cancers (Makuku et al., 2021). In cancer, the tumor cell microenvironment acts to inhibit immune checkpoints, which normally function to prevent uncontrolled proliferation (He and Xu, 2020). Programmed cell death protein 1 (PD-1), an immune checkpoint expressed by several types of immune cells, dampens the immune system upon programmed cell death ligand-1 (PD-L1) binding (Makuku et al., 2021). The interaction of PD-1/PD-L1 leads to the inhibition of phosphorylation of the T-cell-receptor (TCR) signaling intermediate, which terminates the TCR signaling cascade (Keir et al., 2008;Fife et al., 2009). Two signaling motifs in the cytoplasmic tail of PD-1 are the intracellular immunoreceptor tyrosine-based switch motif (ITSM) and the immunoreceptor tyrosine-based inhibitory motif. Upon PD-L1 binding to PD-1, ITSM is phosphorylated and recruits Src homology 2-containing tyrosine phosphatase, thereby inhibiting the phosphatidylinositol 3-kinase (PI3K)/Akt signaling pathway (Barclay et al., 2018;Ai et al., 2020). PI3K/Akt signaling pathway blockage further downregulates the mechanistic targets of rapamycin and inhibits protein synthesis and cell growth. PI3K/Akt signaling pathway blockage also inhibits the degradation of transcription factor FoxO1, which enhances the expression of PD-1 (Barclay et al., 2018;Ai et al., 2020).

The recognition of the PD-1 protein on the membrane of T cells by tumor cells results in the upregulation of PD-L1 (J. Liu et al., 2021). High expression of PD-L1 is one characteristic observed in many types of tumors including melanoma, lung cancer, and breast cancer (Mu et al., 2011; Fusi et al., 2015;Aguilar et al., 2019). PD-1/PD-L1 binding results in T-cell apoptosis (J. Liu et al., 2021). Blockade of the PD-1/PD-L1 axis results in tumor suppression due to interference between the tumor cell and the T cell (C. Liu et al., 2021; Makuku et al., 2021). Numerous studies have demonstrated that the blockage of PD-L1 or PD-1 is one of the most promising approaches for cancer immunotherapy (Zitvogel and Kroemer, 2012; Wu et al., 2018; Salmaninejad et al., 2019). Blocking the interactions of PD-L1 and PD-1 shuts off the inhibitory signaling pathways for T cells, reactivates the T cell–mediated anti-tumor responses by promoting T-cell proliferation, and enhances effector T-cell function (Salmaninejad et al., 2019; Tang and Zheng., 2018). Clinical data have demonstrated that the blockade of PD-1 or PD-L1 can boost T cell–mediated antitumor responses, generates durable clinical responses, and prolongs patient survival time (Ohaegbulam et al., 2015; Alsaab et al., 2017). Monoclonal antibodies against PD-1 (Pembrolizumab, Nivolumab, and Cemiplimab) or PD-L1 (Atezolizumab, Avelumab, and Durvalumab) have been approved by FDA for the treatment of a series of malignancies including breast cancer, bladder cancer, colorectal cancer, lung cancer, hepatoma, and melanoma (Massard et al., 2016;Kim, 2017; Xin Yu et al., 2020). Although these monoclonal antibodies demonstrated promising results with high clinical efficacy and immune-related adverse effects, immunogenicity and high costs are still major limitations of antibody-based immune checkpoint inhibitors (Hamanishi et al., 2015; Alsaab et al., 2017; Zinzani et al., 2017; Akinleye and Rasool et al., 2019). Alternatively, small-molecule inhibitors can overcome these advantages due to better tumor penetration and oral availability (Zhan et al., 2016).

Therefore, the discovery of small molecule inhibitors blocking the PD-1/PD-L1 interaction is a promising cancer therapy approach. Our group reported the evaluation of the PD-1/PD-L1 blockade (using a pair-ELISA technique) and the binding of compounds to either PD-1 or PD-L1 (Li et al., 2022). However, this method is not efficient for large-scale screenings of small molecule libraries for PD-1/PD-L1 inhibitors. Therefore, we developed a surface plasmon resonance (SPR)-based PD-1/PD-L1 blockade screening approach utilizing immobilized PD-1 (on the chip), PD-L1 (in solution), and known inhibitors (i.e., BMS-1166 or BMS-202, in solution). To exclude potential false positives, we included a negative PD-L1 inhibitor (NO-Losartan A) possessing a biphenyl group—a structural feature shared with the BMS-1166 and the BMS-202 compounds that were investigated together in this study. Notably, the SPR technique is a valuable complementary method to ELISA immunoassays that can also be used for the optimization of ELISA-based assays (Vaisocherová et al., 2009). SPR is an optical biosensor technology that employs the evanescent wave phenomenon to detect changes in the refractive index of a biosensor (Pattnaik, 2005). A light source illuminates the biosensor and prism, and as the analyte flows through the channel and binds to the target protein, the refractive index of the biosensor undergoes a shift. This interaction between analyte and protein is monitored in real-time, enabling precise measurement of the amount of bound protein as well as the rates of association and dissociation. The SPR assay has unique advantages over the ELISA-type assay. Rather than merely providing an endpoint, the SPR assay monitors the kinetics associated with the PD-1/PD-L1 blockade of small molecules in real time. We acknowledge that ELISA-type assays are more widely accessible and adaptable to different laboratory settings, and we recognize that our SPR assay requires specialized instrumentation and expertise, which may not be available in all laboratories. Furthermore, given that this blockade assay is solely based on in vitro experiments, it is imperative to perform functional assays and in vivo validation to confirm the potential of compounds exhibiting blockade effects against PD-1/PD-L1. However, we believe that this SPR-based protocol, which provides sufficient details, can facilitate the screening process of small molecule inhibitors that block the PD-1/PD-L1 interaction at a large scale.

In the present study, we utilized an SPR-based assay to determine the IC_50_values of BMS-1166 and BMS-202, which were measured at 85.4 and 654.4 nM, respectively. BMS-1166 has been previously characterized with an IC_50_value of 1.4 and 276 nM by homogeneous time-resolved fluorescence (HTRF) and cell-based assays (Jurkat cells expressing PD-1 in co-culture with CHO cells expressing PD-L1), respectively (Guzik et al., 2017). Previous investigations have reported IC_50_values of BMS-202 at 18 and 96 nM utilizing different assays, including cell-based and HTRF approaches (Surmiak et al., 2021). Our results are comparable with previous findings and confirm the reliability and reproducibility of our SPR-based protocol.

## Materials and reagents

**Biological materials**

1. Human PD-L1/B7-H1 protein, Fc Tag (ACROBiosystems, catalog number: D1-H5258)
2. Human PD-1/PDCD1 protein, Fc Tag, low endotoxin (ACROBiosystems, catalog number: PD1-H5257)

**Reagents**

1. BMS-1166 (Med Chem Express, catalog number: HY-102011)
2. BMS-202 (Med Chem Express, catalog number: HY-19745)
3. Amine Coupling kit [ethanolamine hydrochloride, dimethylaminopropyl-N’ethylcarbodiimide N-3-hydrochloride (EDC), and N-hydroxy succinimide (NDC)] (Global Life Sciences Solutions, Cytiva, catalog number: BR100050)
4. NO-Losartan A (Cayman Chemical Company, catalog number: Cay10006456)

**Solutions**

1. HBS-EP+ buffer 10× (Global Life Sciences Solutions, Cytiva, catalog number: BR100826)
2. Glycine 1.5 (Global Life Sciences Solutions, Cytiva, catalog number: BR100354)
3. Dimethyl sulfoxide (DMSO), anhydrous ≥ 99.9% (Sigma-Aldrich, catalog number: 276855)
4. DNase-free water (Fisher Scientific, catalog number: 188506)
5. Acetate 5.0 (Global Life Sciences Solutions, Cytiva, catalog numbers: BR100350, BR100351)
6. NaOH 50 mM (Global Life Sciences Solutions, Cytiva, catalog number: 100358)
7. PD-1 or PD-L1 protein solution, 500 μg/mL (PD-L1 protein solution equals to 2,000 nM) (see Recipes)
8. HBS-EP+ running buffer (250 mL Fisherbrand glass bottle) (see Recipes)
9. HBS-EP+ running buffer + 0.01% DMSO (250 mL Fisherbrand glass bottle) (see Recipes)

**Recipes**

1. **PD-1 or PD-L1 protein solution, 500 μg/mL (PD-L1 protein solution equals to 2,000 nM)**
  Add 200 μL of DNase-free water to 100 μg of PD-1 or PD-L1 protein. (To prevent nucleic acid contamination, it is recommended to use DNase-free water for the preparation of a long-term stock solution of PD-1 or PD-L1 proteins. However, for assays utilizing fresh protein solutions, Milli-Q water is sufficient.)
2. **HBS-EP+ running buffer (250 mL Fisherbrand glass bottle)**
  Add 20 mL of the HBS-EP+ buffer 10× to 180 mL of Milli-Q water.
3. **HBS-EP+ running buffer + 0.01% DMSO (250 mL Fisherbrand glass bottle)**
  - Add 25 mL of the HBS-EP+ buffer 10× to 224.975 mL of Milli-Q water.
  - Add 25 μL DMSO.

**Laboratory supplies**

1. Pipettes (ErgoOne Single Channel Pipette 2.5, 10, 200, 1,000 μL; USA Scientific, catalog numbers: 7100-0125, 7100-0510, 7100-2200, 7110-1000)
2. Pipette Tips (TipOne 10, 200, 1,000 μL; USA Scientific, catalog numbers: 1111-3800, 1110-1800, 1111-2821)
3. SCI-Fill motorized pipette filler (Scilogex, catalog number: 740200029999)
4. Serological pipettes 10 and 50 mL (Thermo Scientific^TM^Nunc^TM^, catalog numbers: 02-923-204, 02-923-206)
5. 96-well polystyrene microplates (Global Life Sciences Solutions, Cytiva, catalog number: BR100503)
6. Microplate foil, 96-well (Global Life Sciences Solutions, Cytiva, catalog number: 28975816)
7. Plastic vials 7 mm (Global Life Sciences Solutions, Cytiva, catalog number: BR100212)
8. Rubber caps, type 3 (Global Life Sciences Solutions, Cytiva, catalog number: BR100502)
9. Series S Sensor Chip CM5 (Global Life Sciences Solutions, Cytiva, catalog number: BR100530)
10. Fisherbrand reusable glass media bottles with cap 250 mL (Fisher Scientific, catalog number: FB800100)
11. Microcentrifuge tube 1.5 mL non-sterile (Cell Treat, Wilkem Scientific, catalog number: LCEL229441)
12. PCR tubes individual 0.2 mL flat cap (PureAmp, Wilkem Scientific, catalog number: LMTP3030)

## Equipment

1. Biacore T200 SPR (Global Life Sciences Solutions, Cytiva, catalog number: 28975001)

## Software and datasets

1. Biacore T200 analysis software (BIAevaluation version 4.1)
2. GraphPad Prism 9.1.2 (https://www.graphpad.com/updates/prism-912-release-notes)

## Procedure

- **Immobilization of PD-1: Amine Coupling Method**
  1. Set immobilization method on the SPR instrument.
*Note: All steps should follow the instrument’s manual.*
    - Set temperature to 25 °C.
    - Set chip type as CM5.
    - Set flow cells per cycle as 1.
    - Under flow cell 1, check immobilize flow cell 1.
      i. Set amine as the method.
      ii. Set blank immobilization.
    - Under flow cell 2, check immobilize flow cell 2.
      i. Set amine as the method.
      ii. Set ligand as 40 μg/mL PD-1.
      iii. Set aim for immobilization method with desired target level and the wash solution as 50 mM NaOH.
    - Set tubes R2 B1 as 40 μg/mL PD-1.
    - Set tubes R2 B2 as 50 mM NaOH.
    - Set tubes R2 B3 and R2 C3 as Ethanolamine.
    - Set tubes R2 B4 and R2 C4 as Empty.
    - Set tubes R2 B5 and R2 C5 as NHS.
    - Set tubes R2 B6 and R2 C6 as EDC.
  2. Prepare 200 mL of HBS-EP+ running buffer solution (see Recipes).
  3. Prepare Amine Coupling kit reagents and PD-1 protein in a reagent rack.
    - Prepare a stock solution of PD-1 at 500 μg/mL in DNase-free water (see Recipes).
      i. Set at room temperature for 30 min to fully dissolve.
      ii. Dilute PD-1 solution to 40 μg/mL in acetate 5.0 and add 160 μL to tube R2 B1.
    - Add 70 μL of 50 mM NaOH to tube R2 B2.
    - Add 140 μL of ethanolamine to tubes R2 B3 and R2 C3.
    - R2 B4 and R2 C4 remain empty.
    - Add 100 μL of NHS to tube R2 B5 and R2 C5 (NHS is included in the Amine Coupling kit).
    - Add 100 μL of EDC to tubes R2 B6 and R2 C6 (EDC is included in the Amine Coupling kit).
  4. Place Tube A into the HBS-EP+ running buffer solution.
  5. Eject the maintenance sensor chip and insert the CM5 chip.
  6. Reopen the immobilization method, eject the rack, and insert reagent rack 2.
  7. Run method for the estimated run time.
  8. The following method will be performed:
    - Inject ligand solution for five pre-concentrations.
    - Establish a baseline with an injection of the HBS-EP+ running buffer solution.
    - Mix inject a 50:50 ratio of EDC + NHS with a contact time of 420 s and a flow rate of 10 μL/min to activate the chip surface with the modification of carboxymethyl groups to N-hydroxysuccinimide esters.
    - Continue baseline with an injection of the HBS-EP+ running buffer solution after chip modification. The baseline activation will observe a slight response unit (RU) effect.
    - Inject 40 μg/mL of PD-1 ligand to induce an electrostatic interaction that will couple the ligand to the chip surface. The ligand includes both immobilized and non-covalently bound proteins. At this stage, the PD-1 solution remains in contact with the CM5 sensor surface, resulting in a response that includes both immobilized and non-covalently bound PD-1. The N-hydroxysuccinimide esters present on the sensor chip surface react spontaneously with the primary amines on PD-1 to form stable and covalent links.
    - Immobilize the ligand prior to deactivation. This indicates that the ligand has surpassed the protein surface and the majority of the non-covalently bound ligand has been removed.
    - Deactivate remaining NHS-esters and remove unreacted esters through the injection of ethanolamine with NaOH utilizing a contact time of 420 s and a flow rate of 10 μL/min. The unreacted NHS-esters were deactivated using 35 μL of 1 M ethanolamine hydrochloride, which was adjusted to pH 8.5 with NaOH. Additionally, the deactivation process ensures the removal of any remaining electrostatically bound PD-1.
*Notes:*
*i. A slight increase in RU is observed due to the change in the bulk refractive index.*
*ii. Stopping point if desired. Eject chip, gently wash chip surface with two drops of DI water, briefly let dry, and place at 4 °C. Replace running buffer with Milli-Q water for standby. The reproducibility of this immobilization protocol has been confirmed through multiple experiments conducted in our research group. It is important to note that when dissolving PD-L1 recombinant proteins (in lyophilized solid form), a minimum of 30 min of equilibrium time at room temperature is necessary to ensure full dissolution of the protein.*
- **Validation of PD-1/PD-L1 interaction**
  1. Set PD-1/PD-L1 validation method on the SPR instrument.
    - Set temperature to 25 °C.
    - Under General Settings, set the data collection rate at 10 Hz, detection as multi, sample compartment temperature at 25 °C, and concentration unit as nM.
    - Under Assay Steps, set conditioning replicates to 20 times, startup to kinetics with replicates at 10 times, sample to kinetics with 1 replicate, and temperature at 25 °C.
    - Under Cycle Types, select new and enter Kinetics.
      i. In commands, insert Capture, sample 1, and select the sample settings as high performance with a contact time of 120 s, dissociation time of 120 s, a flow rate of 10 μL/min, and a flow path of 1, 2, 3, 4. Under method variables, set property as a variable and select sample solution. Under evaluation variables, select evaluation purpose as kinetics/affinity and select the predefined variables as Conc and MW.
      ii. In commands, insert Regeneration 1 and enter the Regeneration solution as Glycine 1.5 with a contact time of 30 s, a flow rate of 30 μL/min, and a flow path of 1, 2, 3, 4.
      iii. In commands, insert Carry-over control 1 (injection of 30 s with a flow rate of 40 μL/min).
    - Under Cycle Types, select new and enter Conditioning. In commands, select capture and set it as Carry-over control 1 (injection of 30 s with a flow rate of 40 μL/min).
    - Under Variable Settings, select startup and select Define all values in the method. Enter values for the variables as sample buffer, under the sample 1 Sample Solution column header.
    - Under Variable Settings, select a sample and select Define all values at run time. The table should read the Command as Sample, and the Variable as Sample solution, Conc, and MW.
    - Select Verification to ensure the method has been verified and can be used to set up a run.
    - Review Overview assay steps including conditioning, startup, and sample and ensure the settings are as desired.
    - Select Setup Run.
    - Under Detection, select the flow path as 2-1, 4-3.
    - Input the sample solution: PD-L1 with concentrations from 0, 5, 10, 20 to 40 nM and a molecular weight of 51,300 Da.
    - Next, review the overview of assay steps for verification.
    - Select prime before the run.
    - Set each PD-L1 concentration as a separate sample well position in a 96-Well Microplate layout:
      i. R1 A1 as PD-L1 0 nM.
      ii. R1 A2 as PD-L1 5 nM.
      iii. R1 A3 as PD-L1 10 nM.
      iv. R1 A4 as PD-L1 20 nM.
      v. R1 A5 as PD-L1 40 nM.
    - Set Glycine 1.5 for regeneration in the same plate layout.
      i. R1 B1–B12.
    - Set sample buffer for a startup in desired well positions.
      i. R1 C1–C4.
  2. Prepare 200 mL of HBS-EP+ running buffer solution (see Recipes).
  3. Prepare PD-L1 concentrations.
    - Prepare PD-L1 protein at 500 μg/mL (2,000 nM) (see Recipes).
    - Dilute to 40 nM (200 μL) in HBS-EP+ running buffer and add to the 96-well plate (R1 A5).
    - Add 100 μL of HBS-EP+ running buffer (R1 A1, R1 A2, R1 A3, and R1 A4) and perform a 2-fold serial dilution (100 μL) in the plate to 20, 10, and 5 nM.
  4. Add 250 μL of the regeneration solution (Glycine 1.5) to R1 B1–B12.
  5. Add 100 μL of HBS-EP+ running buffer to R1 C1 and 225 μL to R1 C2–C4.
  6. Tightly cover the microplate with a microplate seal.
  7. Remove the previously immobilized PD-1 CM5 chip from 4 °C.
  8. Eject the maintenance chip and insert the PD-1 CM5 chip.
  9. Place Tube A into the HBS-EP+ running buffer + 0.01% DMSO solution.
  10. Open the established method, eject the rack, and insert a 96-well microplate.
  11. Run method for the estimated run time.
  12. After running the method, eject the chip and insert the maintenance chip.
  13. Replace the running buffer with Milli-Q water for standby.
*Note: The reproducibility of the PD-1/PD-L1 interaction step has been confirmed through multiple experiments conducted in our research group. This step is crucial in determining the binding affinity of PD-L1 at different concentrations, which, in turn, is necessary for identifying the optimal concentration of PD-L1 for subsequent blockade assays.*
- **PD-1/PD-L1 blockade assay with established small molecule inhibitor: BMS-1166**
  1. Set the method on the SPR instrument.
    - Set temperature to 25 °C.
    - Set General Settings as the same as the validation protocol.
    - Under Assay Steps, set conditioning replicates to 20 times, set startup to conditioning with replicates at 10 times, sample to kinetics with 1 replicate, and temperature at 25 °C.
    - Set Cycle Types (both kinetics and conditioning) as the same as the validation protocol.
    - Under Variable Settings, select startup and select Define all values in the method. Enter values for the variables as sample buffer, under the sample 1 Sample solution column header.
    - Select Verification to ensure the method has been verified and can be used to set up a run.
    - Review Overview assay steps including conditioning, startup, and sample, and ensure the settings are as desired.
    - Select Setup Run.
    - Under Detection, select the flow path as 2-1, 4-3.
    - Input the sample solution: PD-L1 20 nM + BMS-1166 with concentrations from 0, 1, 5, 25, 125, 625, to 3,125 nM and a molecular weight of 51,300 Da.
    - Next, review the overview of assay steps for verification.
    - Select prime before run.
    - Set each PD-L1/BMS-1166 concentration as a separate sample well position in a 96-well microplate layout:
      i. R1 A1 as PD-L1 20 nM + BMS-1166 0 nM.
      ii. R1 A2 as PD-L1 20 nM + BMS-1166 1 nM.
      iii. R1 A3 as PD-L1 20 nM + BMS-1166 5 nM.
      iv. R1 A4 as PD-L1 20 nM + BMS-1166 25 nM.
      v. R1 A5 as PD-L1 20 nM + BMS-1166 125 nM.
      vi. R1 A6 as PD-L1 20 nM + BMS-1166 625 nM.
      vii. R1 A7 as PD-L1 20 nM + BMS-1166 3,125 nM.
    - Set Glycine 1.5 for regeneration in reagent rack 2.
      i. R2 A1.
  2. Prepare 250 mL of HBS-EP+ running buffer solution (see Recipes).
  3. Prepare PD-L1 solution at 20 nM in HBS-EP+ running buffer (1.5 mL microcentrifuge tube).
    - Add 1.5 μL of PD-L1 protein solution (2,000 nM stock to 148.5 μL of HBS-EP+ running buffer).
  4. Prepare PD-L1 solution at 20 nM in HBS-EP+ running buffer + 0.01% DMSO (1.5 mL microcentrifuge tube).
    - Add 5 μL of PD-L1 protein solution 2,000 nM stock to 494.5 μL of HBS-EP+ running buffer.
    - Add 0.5 μL of 10% DMSO.
  5. Prepare the BMS-1166 concentrations and the regeneration solution in a 96-well microplate.
    - Prepare a 31.25 mM stock of BMS-1166 in DMSO (10 μL) (in PCR tube).
    - Dilute the BMS-1166 stock to 3,125 nM in 100 μL of PD-L1 (20 nM) HBS-EP+ running buffer and add to the 96-well plate: R1 A7.
    - Add 80 μL of PD-L1 (20 nM) + HBS-EP+ running buffer + 0.01% DMSO solution to R1 A6–R1 A2 and perform a 5-fold serial dilution (20 μL) in the plate to 625, 125, 25, 5, and 1 nM.
    - Add 80 μL of PD-L1 (20 nM) + HBS-EP+ running buffer + 0.01% DMSO solution to R1 A1.
    - Add 3 mL of the regeneration solution (Glycine 1.5) to R2 A1.
  6. Add 0.01% DMSO to HBS-EP+ to use as the running buffer.
  7. Tightly cover the plate with a microplate seal.
  8. Remove the previously immobilized PD-1 CM5 chip from 4 °C.
  9. Eject the maintenance chip and insert PD-1 CM5 chip.
  10. Place Tube A into the HBS-EP+ running buffer + 0.01% DMSO solution.
  11. Open the established method, eject the rack, and insert the 96-well microplate.
  12. Run method for the estimated run time.
  13. After running the method, eject the chip and insert the maintenance chip.
  14. Wash the chip with two drops of DI water and place at 4 °C.
  15. Replace running buffer with Milli-Q water for standby.
- **PD-1/PD-L1 blockade assay with established small molecule inhibitor: BMS-202**
  1. Set the same method on the SPR instrument as the BMS-1166 method.
    - Set each PD-L1/BMS-202 concentration as a separate sample well position in a 96-well microplate layout:
      i. R1 A1 as PD-L1 20 nM + BMS-202 0 nM.
      ii.R1 A2 as PD-L1 20 nM + BMS-202 10 nM.
      iii. R1 A3 as PD-L1 20 nM + BMS-202 100 nM.
      iv. R1 A4 as PD-L1 20 nM + BMS-202 500 nM.
      v. R1 A5 as PD-L1 20 nM + BMS-202 1,000 nM.
      vi. R1 A6 as PD-L1 20 nM + BMS-202 5,000 nM.
    - Set Glycine 1.5 for regeneration in reagent rack 2.
      i. R2 A1.
  2. Prepare 250 mL of HBS-EP+ running buffer.
  3. Prepare PD-L1 solution (20 nM) in HBS-EP+ running buffer (1.5 mL microcentrifuge tube).
    - Add 1.5 μL of PD-L1 protein solution (2,000 nM stock to 148.5 μL of HBS-EP+ running buffer).
  4. Prepare PD-L1 solution (20 nM) in HBS-EP+ running buffer + 0.01% DMSO solution (1.5 mL microcentrifuge tube).
    - Add 5 μL of PD-L1 2,000 nM stock to 494.5 μL of HBS-EP+ running buffer.
    - Add 0.5 μL of 10% DMSO.
  5. Prepare the BMS-202 concentrations and the regeneration solution in a 96-well microplate.
    - Prepare 50 mM stock solution of BMS-202 dissolved in DMSO.
    - In R1 A6, dilute the BMS-202 stock to 5,000 nM in 120 μL of PD-L1 solution (20 nM) in HBS-EP running buffer.
    - In R1 A5, perform a 5-fold dilution by adding 20 μL from R1 A6 with 80 μL of PD-L1 solution (20 nM) in HBS-EP+ running buffer + 0.01% DMSO.
    - In R1 A4, perform a 10-fold dilution by adding 10 μL from R1 A6 with 90 μL of PD-L1 solution (20 nM) in HBS-EP+ running buffer + 0.01% DMSO.
    - In R1 A3, perform a 10-fold dilution by adding 10 μL from R1 A5 with 90 μL of PD-L1 solution (20 nM) in HBS-EP+ running buffer + 0.01% DMSO.
    - In R1 A2, perform a 10-fold dilution by adding 10 μL from R1 A3 with 90 μL of PD-L1 solution (20 nM) in HBS-EP+ running buffer + 0.01% DMSO.
    - In R1 A1, add 90 μL of PD-L1 protein solution (20 nM) in HBS-EP+ running buffer + 0.01% DMSO.
    - Add 3 mL of the regeneration solution (Glycine 1.5) to R2 A1.
    - Add 0.01% DMSO to the HBS-EP+ running buffer.
  6. Proceed with the same procedure as BMS-1166 to run BMS-202.
- **PD-1/PD-L1 blockade assay with negative control: NO-Losartan A**
  1. Set the same method on the SPR machine as the BMS-1166/BMS-202 methods.
    - Set each PD-L1/NO-Losartan A concentration as a separate sample well position in a 96-well microplate layout:
      i. R1 A1 as PD-L1 20 nM + NO-Losartan A 0 nM.
      ii. R1 A2 as PD-L1 20 nM + NO-Losartan A 1 nM.
      iii. R1 A3 as PD-L1 20 nM + NO-Losartan A 5 nM.
      iv. R1 A4 as PD-L1 20 nM + NO-Losartan A 25 nM.
      v. R1 A5 as PD-L1 20 nM + NO-Losartan A 125 nM.
      vi. R1 A6 as PD-L1 20 nM + NO-Losartan A 625 nM.
      vii. R1 A7 as PD-L1 20 nM + NO-Losartan A 3,125 nM.
  2. Prepare PD-L1 (20 nM), NO-Losartan at different concentrations, HBS-EP+ running buffer, HBS-EP+ running buffer +0.01% DMSO buffer, and regeneration solution, as described in the BMS-1166 protocol.
  3. Proceed with the same procedure as BMS-1166/BMS-202 to run NO-Losartan A.
*Note: In this protocol, NO-Losartan A was employed as a negative control due to the presence of a biphenyl group, which is a structural feature shared by the BMS-1166 and BMS-202 compounds tested. To ascertain the suitability of NO-Losartan A as a negative control, we conducted a preliminary investigation of its binding affinity with PD-L1. Our findings indicated that NO-Losartan A displayed negligible binding affinity towards PD-L1, confirming its suitability as a negative control in this protocol. For screening purposes, the selection of a negative control may depend on the specific composition of the compound library employed. It is essential to ensure that the chosen negative control exhibits no binding affinity towards the PD-L1.*

## Data analysis

1. Immobilization of PD-1:
  Data were analyzed via the output from the SPR instrument indicating a low RU of the blank cell and a successful target RU of the PD-1 ligand.
2. Validation of PD-1/PD-L1 interaction:
  Data were analyzed using the corresponding Biacore T200 analysis software (BIAevaluation version 4.1)
  i. Under kinetics/affinity, select surface bound.
  ii. Select the curve as 2-1.
  iii. Perform a 1:1 binding model with a constant fit to obtain the association rate (K_a_), dissociation rate (K_d_), and dissociation constant (K_D_).
  iv. Export analyzed curves into GraphPad Prism for graphical representation of data.
3. PD-1/PD-L1 blockade assay with established small molecule inhibitors (BMS-1166 and BMS-202) and negative control (NO-Losartan A):
  - Data were analyzed using the corresponding Biacore T200 analysis software.
  - Under kinetics/affinity, select surface bound.
  - Select the curve as 2-1.
  - Export curves into GraphPad Prism for graphical representation of data.
  - Blockade rate and IC_50_value of each compound were analyzed using GraphPad Prism.
  - To determine the percentage blockade of each sample concentration, employ the subsequent formula: Percentage blockade (%) = [1 – (RU of PD-L1 incubated with the compound/RU of PD-L1 in the absence of the compound)] × 100.
  - The concentration of each compound and the blockade rate of each compound were imported into GraphPad Prism.
  - The XY analysis function was selected, and log (inhibitor) vs. response – Variable slope (four parameters) was chosen.
  - The IC_50_values of each compound were obtained, and a goodness-of-fit assessment was performed. A coefficient of determination (R-squared) greater than 0.99 was required for a satisfactory fit.

## Validation of protocol

1. Immobilization of PD-1 on SPR chip:
  - Flow cell 1 was immobilized as the blank with a final response (RU) of 103.1 (Figure 1A).
  - Flow cell 2 was immobilized with PD-1 ligand coated on the chip surface with a final response (RU) of 3688.5, indicating a successful reach of the target (Figure 1B).
    **Figure 1.**
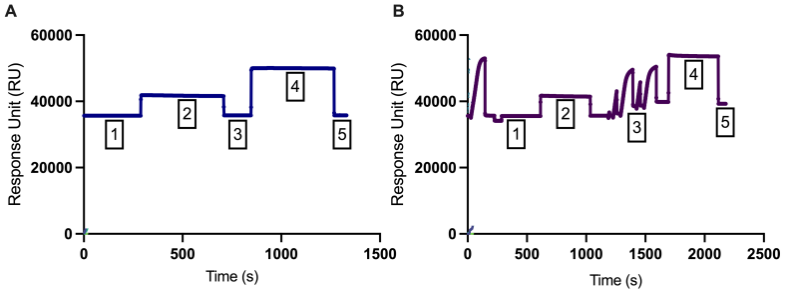
Immobilization curves of (A) flow cell 1 (blank) and (B) flow cell 2 (PD-1). The first phase (phase 1) represents a stable baseline, while the second phase (phase 2) displays a responsive effect in the response unit (RU). In phase 3, a wash with ethanolamine hydrochloride (1 M; pH 8.5) was conducted. In phase 4, recombinant PD-1 protein (40 μg/mL) was injected and coupled to the surface matrix of flow cell 2, and running buffer was injected in the flow cell 1. In phase 5, any remaining electrostatically bound ligand was removed using ethanolamine hydrochloride (1 M; pH 8.5) to deactivate unreacted NHS-esters with a contact time of 420 s and a flow rate of 10 μL/min.
2. Validation of PD-1/PD-L1 interaction:
  - The binding interaction of 5, 10, 20, and 40 nM PD-L1 in solution to PD-1 on the chip surface was observed with a quantifiable response (Figure 2).
  - The analyzed binding parameters included an association rate (K*_on_*) = 8.852 × 10^4^1/Ms, dissociation rate (K*_off_*) = 0.01146 1/s, and dissociation constant (K*_D_*) = 1.295 × 10^-7^M.
    **Figure 2.**
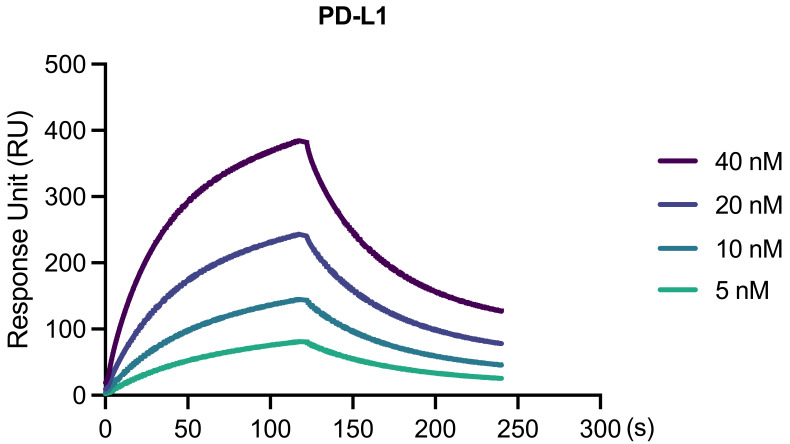
Binding kinetics of PD-L1 to PD-1 at varying concentrations. The real-time surface plasmon resonance (SPR) response of the sensor chip to the binding reactions between PD-L1 (in solution) and PD-1 (immobilized on the chip) is displayed. The concentration series of PD-L1 used (5–40 nM; 2-fold dilutions) is shown. PD-L1 demonstrates distinct association (0–120 s) and dissociation phases (121–240 s) with PD-1, which are clearly visible.
3. PD-L1/PD-1 blockade assay with established small molecule inhibitor: BMS-1166
  Blockade of the PD-1/PD-L1 binding interaction was observed by 1–3,125 nM BMS-1166 with 20 nM PD-L1 protein in 0.01% DMSO solution (Figure 3A). There was a concentration-dependent increase in percentage blockade of PD-1/PD-L1 with an IC_50_of 85.4 nM (Figure 3B). The percentage blockade for 0, 1, 5, 25, 125, 625, and 3,215 nM BMS-1166 was 0%, 12.7%, 18.5%, 31.5%, 62.4%, 85.9%, and 94.2%, respectively (Figure 3C).
  **Figure 3.**
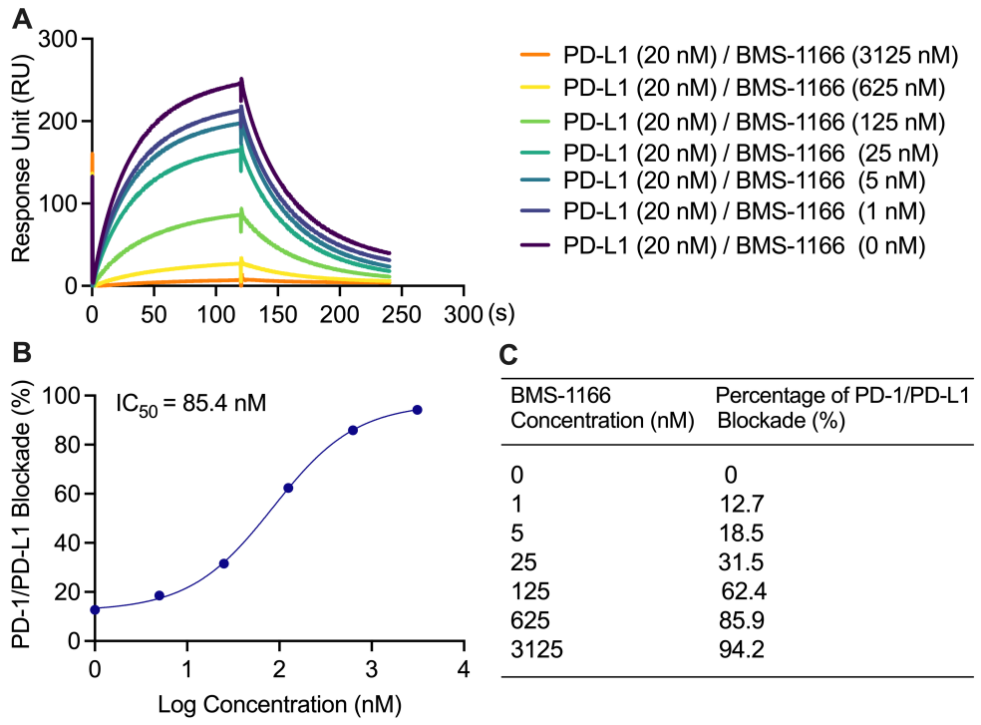
Blockade of PD-1/PD-L1 interaction with BMS-1166. (A) A representative real-time surface plasmon resonance (SPR) response to the binding reactions between PD-L1 (in solution) and PD-1 (immobilized on the chip) in the presence of BMS-1166, at various concentrations ranging from 1 to 3,215 nM, with a fixed concentration of 20 nM of PD-L1 in solution. (B) The sigmoidal binding profile of BMS-1166 on the log scale with an indicated IC_50_of 85.4 nM. (C) BMS-1166 concentration-dependent increase in percentage blockade.
4. PD-1/PD-L1 blockade assay with established small molecule inhibitor: BMS-202:
  Blockade of the PD-1/PD-L1 binding interaction was observed by 1–5,000 nM BMS1166 with 20 nM PD-L1 protein in a solution with 0.01% DMSO (Figure 4A). There was a concentration-dependent increase in percentage blockade of PD-1/PD-L1 with an IC_50_of 654.4 nM (Figure 4B). The percentage blockade for 0, 100, 500, 1,000, 5,000, 5,000, and 50,000 nM BMS-202 was 0%, 14.9%, 18.5%, 32.0%, 56.4%, and 67.1%, respectively (Figure 4C).
  **Figure 4.**
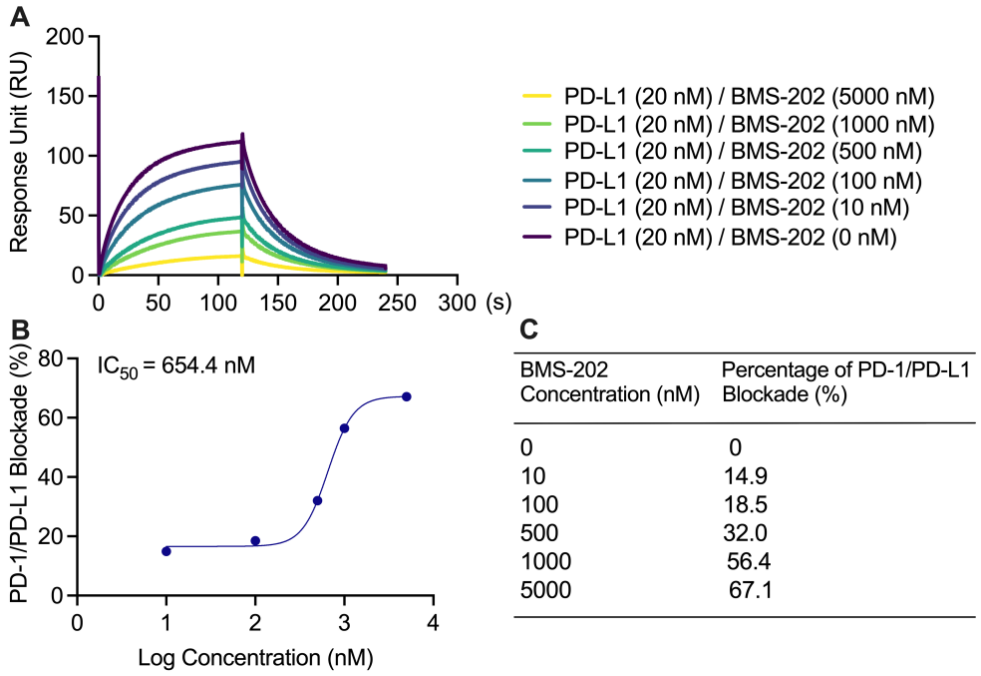
Blockade of PD-1/PD-L1 interaction with BMS-202. (A) BMS-202 at 0, 10, 100, 500, 1,000, and 5,000 nM with 20 nM PD-L1 in solution lowered the PD-1/PD-L1 binding response unit (RU), concentration dependently. (B) The sigmoidal binding profile of BMS-202 on the log scale with an indicated IC_50_of 654.4 nM. (C) BMS-202 concentration-dependent increase in percentage blockade.
5. PD-1/PD-L1 blockade assay with negative control: NO-Losartan A:
  - No blockade of the PD-1/PD-L1 interaction was observed at 0, 1, 5, 25, 125, 625, and 3,125 nM NO-Losartan A (negative control) with 20 nM PD-L1 in 0.01% DMSO solution (Figure 5A).
  - The percentage blockade for 0, 1, 5, 25, 125, 625, and 3,215 nM NO-Losartan A was 0%, 1.08%, 1.51%, 1.21%, 2.05%, 0%, and 0%, respectively (Figure 5B).
    **Figure 5.**
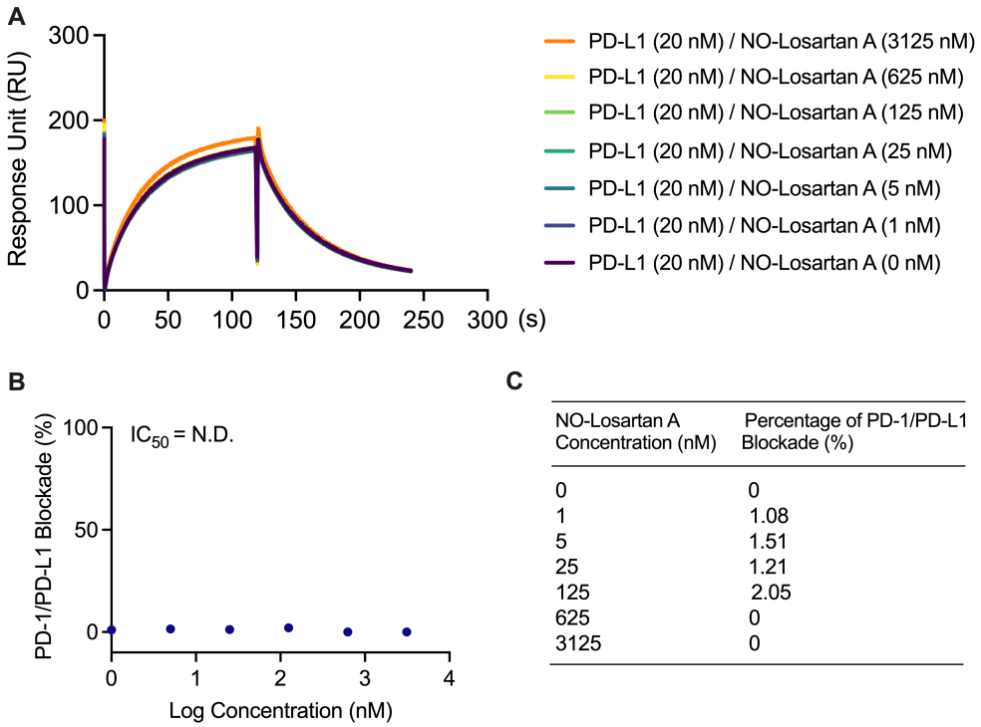
Failure to block the PD-1/PD-L1 interaction with NO-Losartan A. (A) PD-L1 was incubated with increasing concentrations of NO-Losartan A (0, 1, 5, 25, 125, 625, and 3,125 nM). The binding between PD-1 and PD-L1 was then assessed using a surface plasmon resonance (SPR) assay. (B) The inhibitory concentration (IC_50_) of NO-Losartan A could not be determined, as it failed to inhibit the PD-1/PD-L1 interaction. (C) Percentage of PD-1/PD-L1 interaction blockade by NO-Losartan A at all concentrations tested.

## General notes and troubleshooting

**General notes**

1. This protocol outlines the procedure for the Biacore T200 system, which requires BIA evaluation software (version 4.1). While other SPR platforms from different brands can also be used, it should be noted that the specific steps may vary depending on the system’s operation. Key steps in this protocol include immobilizing a sufficient amount of PD-1 (more than 3500 RU) on the chip and running PD-L1 solution with a series of concentrations to obtain an observable RU.
2. Ensure there are no bubbles in any of the tubes. If there are bubbles, sonication can be performed to remove them.
3. Each control, BMS-1166, BMS-202, and NO-Losartan A were run separately. However, when running samples, they can be run in the same experiment as positive and negative controls. Furthermore, we evaluated the IC_50_values of BMS-1166 (20–10,000 nM) and BMS-202 (20–10,000 nM) and found that they demonstrated similar IC_50_values in Figure S1. Specifically, the IC_50_value for BMS-1166 was 80.47 nM and the IC_50_value for BMS-202 was 359.1 nM.
4. Technical replicates can be useful in this protocol; we recommend carefully considering the experimental design and the potential sources of variability when using SPR to investigate the blockade rates of different compounds using proteins from different sources. Depending on the nature of the experiment and the biological variability of the samples, it may be appropriate to include biological replicates to ensure accurate and reliable results.
5. The concentration of PD-L1 recombinant protein can vary depending on the RU. Although 20 nM of PD-L1 was used in this protocol and displayed an observable RU, a lower concentration may still be useful. However, it is not recommended to use PD-L1 with a concentration that leads to a RU of less than 10 RU, as this may result in an unfavorable signal-to-noise ratio. In addition, conducting technical replicates for this binding kinetics assay may not be necessary, as it is primarily used to confirm the suitability of the concentrations of PD-L1 for use in further blockade assays.
6. Use a final concentration of 0.01% DMSO, as this had the lowest percentage blockade effect on PD-1/PD-L1 binding (Table S1).
7. DMSO with different percentages significantly affected PD-L1 activities, as supported by the data displayed in the Supplementary materials. Try to use a minimum concentration of DMSO (e.g., 0.01% or lower) in the sample preparation and running buffer. Ensure the concentration of DMSO in the sample preparation and running buffer are equal to avoid buffer mismatch effects.

**Troubleshooting**

1. Unsuccessful immobilization can result from either the failure to immobilize PD-1 or a low final RU on the chip. This may be due to a low stock concentration of PD-1, as using a stock solution with a low concentration of PD-L1 in DNase-free water can affect the pH value of the working solution when PD-1 is dissolved in acetate 5.0.
  - Adjust the target response level (increasing it to 5,000 RU).
  - Change the running buffer or protein buffer (preparing a high concentration of PD-1 stock solution up to 1 mg/mL in DNase-free water).
  - Change to a different SPR chip (CM5 chip is used in this study, but the users can try the CM7 chip with a higher loading capacity to increase the amount of PD-1 on the chip).
2. Unsuccessful PD-1/PD-L1 interaction can result from an unsuccessful immobilization or a narrow concentration range of PD-L1.
  a. Vary the protein concentrations (expand the PD-L1 concentration from nM to mM with a 10-fold dilution factor. This can help to identify the optimal concentration range for successful PD-1/PD-L1 interaction. Additionally, if unsuccessful immobilization is the cause, follow the steps outlined in the previous answer to address this issue).

## Supplementary information

The following supporting information can be downloaded here:

1. Figure S1. Inhibition of PD-1/PD-L1 interaction by BMS-202 and BMS-1166
2. Table S1. Effects of DMSO 0, 0.01, 0.05, 0.1, 0.5, and 1% on PD-1/PD-L1 Binding
3. Supplementary materials
